# Enhanced Mitogenic Activity of Recombinant Human Vascular Endothelial Growth Factor VEGF_121_ Expressed in *E*. *coli* Origami B (DE3) with Molecular Chaperones

**DOI:** 10.1371/journal.pone.0163697

**Published:** 2016-10-07

**Authors:** Ondřej Kaplan, Jana Zárubová, Barbora Mikulová, Elena Filová, Jiřina Bártová, Lucie Bačáková, Eduard Brynda

**Affiliations:** 1 Institute of Macromolecular Chemistry, Czech Academy of Sciences, CZ-162 06, Prague, Czech Republic; 2 Institute of Physiology, Czech Academy of Sciences, CZ-142 20, Prague, Czech Republic; 3 Faculty of Science, Charles University in Prague, CZ-128 40, Prague, Czech Republic; 4 School of Dental Medicine, General University Hospital in Prague, CZ-128 08, Prague, Czech Republic; Yale University School of Medicine, UNITED STATES

## Abstract

We describe the production of a highly-active mutant VEGF variant, α_2_-PI_1-8_-VEGF_121_, which contains a substrate sequence for factor XIIIa at the aminoterminus designed for incorporation into a fibrin gel. The α_2_-PI_1-8_-VEGF_121_ gene was synthesized, cloned into a pET-32a(+) vector and expressed in *Escherichia coli* Origami B (DE3) host cells. To increase the protein folding and the solubility, the resulting thioredoxin-α_2_-PI_1-8_-VEGF_121_ fusion protein was co-expressed with recombinant molecular chaperones GroES/EL encoded by independent plasmid pGro7.

The fusion protein was purified from the soluble fraction of cytoplasmic proteins using affinity chromatography. After cleavage of the thioredoxin fusion part with thrombin, the target protein was purified by a second round of affinity chromatography. The yield of purified α_2_-PI_1-8_-VEGF_121_ was 1.4 mg per liter of the cell culture. The α_2_-PI_1-8_-VEGF_121_ expressed in this work increased the proliferation of endothelial cells 3.9–8.7 times in comparison with commercially-available recombinant VEGF_121_. This very high mitogenic activity may be caused by co-expression of the growth factor with molecular chaperones not previously used in VEGF production. At the same time, α_2_-PI_1-8_-VEGF_121_ did not elicit considerable inflammatory activation of human endothelial HUVEC cells and human monocyte-like THP-1 cells.

## Introduction

Therapeutic angiogenesis is a promising approach for treating patients with cardiovascular diseases, and is also critical in engineering vascularized tissue replacements. Vascular endothelial growth factor (VEGF) plays an essential role in regulating normal and pathological angiogenesis and vascular permeability. VEGF promotes the adhesion and growth of vascular endothelial cells, which can be used advantageously for endothelialization of cardiovascular implants, such as small-diameter vascular replacements [1] or endovascular stent grafts [2], and for vascularization of various three-dimensional porous scaffolds for tissue engineering [3]. VEGF-A is one of five members of the VEGF family, along with VEGF-B, VEGF-C, VEGF-D, and placental growth factor (PlGF) [4–7].

The VEGF-A gene consists of a 14-kb coding region organized in eight exons separated by seven introns [8]. The first four exons encode the signal peptide, the sequences of recognition by the VEGF receptors, and the dimerization and glycosylation site. Exon 5 encodes a sequence of ten amino acids that contains the main site of cleavage by plasmin and matrix metalloproteinases [9]. Exons 6 and 7 encode two heparin-binding domains [10]. Due to alternative exon splicing, a large number of VEGF isoforms exist. The most notable in humans are VEGF_121_, VEGF_165_ and VEGF_189_. The number indicates the amino acids in the mature polypeptide after removal of the signal sequence [11]. These isoforms are distinguished by the presence or absence of the peptides encoded by exons 6 and 7. VEGF_121_ lacks both heparin-binding domains, and is therefore diffusible. VEGF_165_ lacks exon 6. VEGF_189_ contains both heparin-binding domains, and is tightly associated with the cell surface or the extracellular matrix [12]. VEGF_165_ and VEGF_189_ might be released from the extracellular matrix (ECM) by plasmin, both directly through digestion of the components of the basement membrane and indirectly by activating collagenases from zymogens [12, 13]. Cleavage of VEGF_165_ and VEGF_189_ by plasmin results in VEGF_110_ [12], which is highly diffusible. VEGF_110_ is biologically and biochemically similar to alternatively spliced VEGF_121_ [14].

The impact of different VEGF-A isoforms on the development and patterning of the vascular system has also been supported by genetic studies using isoform-specific knockouts in mice. Fifty percent of the mice expressing exclusively VEGF_120_ (mouse VEGF-A is one amino acid shorter than human VEGF-A) died soon after birth, and showed impaired cardiac performance and myocardial angiogenesis [15]. VEGF_120/120_ mouse embryos also exhibited a specific decrease in capillary branching, which was probably caused by changes in the extracellular localization of VEGF-A. Endothelial cells were preferentially integrated within existing vessels to increase the lumen caliber, rather than being recruited into newly-formed branches. However, mice expressing only VEGF_188_ displayed abnormally thin vessel branches. Half of the mice died between embryonic stage E9.5 and E13.5 [16, 17]. Mice expressing only VEGF_164_ had no abnormalities [18, 19]. The angiogenic activity of VEGF-A in tissues is thus regulated by different affinity of VEGF-A isoforms from the ECM, and by processing of VEGF-A and ECM by proteases and heparinases. Longer forms of VEGF-A might represent a storage form of the growth factor that is released after degradation of the ECM, while the diffusible forms play a more dynamic role and are readily available to endothelial cells [20, 21].

Due to the critical role of VEGF-A in promoting neovascularization, clinical trials have investigated the administration of VEGF-A as a recombinant protein or gene [22]. The results of phase I trials using intravenous or intracoronary infusions were typically encouraging [23–26]. However, the results obtained in the larger phase II trials failed to prove that there was a significantly more beneficial effect than for the placebo group [27]. These disappointing clinical results have been attributed in part to the biological half-life of intravenously administrated VEGF-A, which is less than 30 min [28]. Owing to the high instability of the protein, large doses and multiple injections would be needed, but these might lead to pathological vessel formation at non-target sites [29]. This demonstrated the need to optimize the delivery method for VEGF-A. One way to improve the delivery and the stability of VEGF-A in the body may be by immobilizing the growth factor into a polymer matrix, where the release can be controlled by the degradation rate of the polymer.

In this study, we prepared a mutant variant α_2_-PI_1-8_-VEGF_121_, first designed by Zisch et al. [30], containing a substrate sequence (NQEQVSPL) for factor XIIIa that would enable covalent incorporation of VEGF_121_ into the fibrin network. VEGF-loaded fibrin matrices have been shown to increase the growth activity of vascular endothelial cells [30]. They can therefore be used for coating vascular prostheses or other cardiovascular implants (stents, heart valve replacements) in order to accelerate their endothelialization. To improve its folding and solubility, the protein was expressed in fusion with thioredoxin. Linkage of the gene of interest to a second 'carrier' or 'partner' gene avoids the problems associated with heterologous protein expression in *E*. *coli* [31, 32]. Glutathione-S-transferase, maltose-binding protein, thioredoxin and NusA have been successful in producing correctly folded and soluble recombinant proteins in bacteria [32, 33].

Thioredoxin (Trx) has been shown to facilitate the soluble expression of a number of mammalian growth factors and cytokines [34]. It is a small, ubiquitous protein that is involved in many physiological functions, and acts both intra- and extracellularly [35]. It works as an important antioxidant that plays a key role in maintaining the reducing environment in the cells. Outside the cell, however, thioredoxin acts as a growth factor or cytokine and stimulates angiogenesis [35–37]. In mammalian cells, thioredoxin is encoded by two Trx genes. The Trx1 isoform occurs in the cytosol and nucleus, while the Trx2 isoform is expressed in the mitochondria. Homozygous knock-out of either isoform in mouse was found to be lethal [37]. Thioredoxin of *E*. *coli* encoded by the TrxA gene is a single polypeptide chain composed of 109 amino acid residues with a molar weight of 11.7 kDa, and is structurally related to human thioredoxin.

Besides fusion with thioredoxin, a combination of pET-32a(+) vector and *E*. *coli* Origami B(DE3) host cells was chosen to increase the soluble protein fraction. This combination was used for several proteins that were difficult to express with a structure related to VEGF [38–40].

In the present study, protein folding was encouraged by co-expression with recombinant molecular chaperones GroES/EL encoded by independent plasmid pGro7. The α_2_-PI_1-8_-VEGF_121_ prepared by the strategy presented here had a 3.9–8.7 times greater effect on endothelial cell proliferation than commercially-available VEGF_121_. Since the final α_2_-PI_1-8_-VEGF_121_ preparation probably contained a small amount of thrombin, which was used for cleavage of the fusion partner and which is able to stimulate the proliferation of endothelial cells [41–43], the effect of appropriate concentrations of thrombin on endothelial cell proliferation were also observed. In addition, VEGF can cause immune activation of cells, which can even lead to implant rejection [44, 45]. We therefore also tested the potential of VEGF to induce the production of pro-inflammatory cytokines and chemokines in human vascular endothelial cells and human monocyte-like THP cells.

## Materials and Methods

### Materials

pET-32a(+) plasmid and *E*. *coli* Origami B (DE3) host cells were obtained from Merck KGaA, Germany. Chaperone plasmid pGro7 was supplied by Takara Bio Inc., Japan. Talon Metal Affinity Resin was purchased from Clontech, USA. Recombinant vascular endothelial growth factor VEGF_121_ variants expressed in *E*. *coli* (VEGF_121_ **I**; Cat. No. CYT-343) and in mammalian HEK cells (VEGF_121_ **II**; Cat. No. CYT-116) came from Prospecbio, USA. The VEGF_121_-ELISA Kit was purchased from Invitrogen, USA. A Cell Proliferation Kit II (XTT) was obtained from Roche, Switzerland. Human Umbilical Vein Endothelial Cells (HUVECs), EBM-2 Basal Medium and EGM-2 SingleQuots were supplied by Lonza, Czech Republic. Concentration cell Amicon Ultra (cut-off 10 kDa) and membrane filters (pore size 0.22 μm) were from Millipore, USA. Dialysis membrane Spectra/Por 6 (MWCO: 1000) was obtained from Spectrum Labs, USA. Coomassie Brilliant Blue R-250 was purchased from Serva, USA. Bradford reagent was from Bio-Rad, Germany. The SDS-PAGE molecular weight protein marker was supplied by GE Healthcare, UK. L-Arabinose, the Resazurin-based In Vitro Toxicology Assay Kit, human monocyte-like THP-1 cells, RPMI-1640 medium, lipopolysaccharides from *E*. *coli* 026:B6 and thrombin from human plasma were purchased from Sigma-Aldrich, Germany.

### Construction of α_2_-PI_1-8_-VEGF_121_ expression vector

The gene sequence encoding the modified variant of human VEGF_121_ (α_2_-PI_1-8_-VEGF_121_), which contains the additional factor XIIIa substrate sequence NQEQVSPL [30] at the aminoterminus of mature VEGF_121_, was synthesized by Generay Biotech, China, according to the published sequences (GenBank). The codon bias was optimized with respect to the *E*. *coli* preferred codon usage. The gene was subcloned between restriction sites *Msc* I and *Xho* I of the expression vector pET-32a(+) after the thioredoxin gene. The nucleotide sequence of the final construct (pET32-VEGF_121_) was confirmed by DNA sequencing.

### Expression of Trx-α_2_-PI_1-8_-VEGF_121_

The construct was used to transform *E*. *coli* Origami B (DE3) competent cells. The strain was co-transformed with the expression vector pGro7 encoding GroEL/ES chaperone genes. *E*. *coli* Origami B (DE3) cells were grown in 2YT medium (10 g of yeast extract, 16 g of tryptone, and 5 g of NaCl per liter; 200 mL in 500-mL Erlenmeyer flasks) with ampicillin (100 mg/L) and chloramphenicol (35 mg/L) in an orbital shaker for ca. 3 h at 37°C and at 220 rpm. When the OD at 600 nm reached a value of ca 0.5, isopropyl-β-D-thiogalactopyranoside (IPTG) was added to a final concentration of 0.02 mM, and the temperature was lowered to 25°C. The expression of GroEL/ES was induced by arabinose (1.7 g/L), which was added at the same time point as IPTG. After 20 h of incubation, the cells were washed with the buffer (50 mM Tris, pH 7.4, 100 mM NaCl, 2.5 mM CaCl_2_), pelleted by centrifugation, and the pellets were stored frozen at -80°C until required.

### Recombinant α_2_-PI_1-8_-VEGF_121_ purification

The harvested cells were then disrupted by sonication, and the cell debris was removed by centrifugation (13 000 rpm, 25 min, 4°C). The supernatant was loaded onto a Talon Metal Affinity Resin column equilibrated with the same buffer. After washing with washing buffer (50 mM Tris, pH 7.4, 100 mM NaCl, 2.5 mM CaCl_2_, 1 mM imidazole), the recombinant protein was eluted with the same buffer containing 200 mM imidazole. The purified fusion protein Trx-α_2_-PI_1-8_-VEGF_121_ was dialyzed against Tris buffer to remove imidazole, and then it was cleaved by human thrombin (9.5 NIH units/ mg of fusion protein, 4 h, RT). The cleaved thioredoxin fusion part was removed by a Talon Metal Affinity Resin column equilibrated with Tris buffer. The purified α_2_-PI_1-8_-VEGF_121_ was analyzed by 12% SDS-PAGE followed by Coomassie Brilliant Blue staining.

### Cell culture

Human Umbilical Vein Endothelial Cells (HUVECs) were cultured in EBM-2 basal medium (Lonza, Cat. No. CC-3156), supplemented with EGM-2 SingleQuots (Lonza, Cat. No. CC-4176) containing 2% fetal bovine serum in a humidified air atmosphere with 5% CO_2_ at 37°C.

### Endothelial cell proliferation followed by the xCELLigence system

The effect of purified VEGF_121_ on proliferation of the endothelial cells was evaluated by the xCELLigence System (Roche Applied Science). This device consists of microtiter plates containing interdigitated gold microelectrodes. This enables label-free, real-time monitoring of cell growth and viability based on electrical impedance measurements.

First, the E-plate background signal corresponding to the culture medium was set up. The E-plate was seeded with HUVEC cells (3 500 per well) in 150 μL EBM-2 basal medium supplemented with ascorbic acid, hydrocortisone, heparin, gentamicin, amphotericin-B and 2% fetal bovine serum from EGM-2 SingleQuots and different concentrations of recombinant α_2_-PI_1-8_-VEGF_121_ (20, 50, and 100 ng/mL) or commercial VEGF_121_ **I** and VEGF_121_ **II** as standards (20, 50, and 100 ng/mL). The hEGF, VEGF, R3-IGF-1 and h-FGF-beta that are also present in the SingleQuots supplement were not added into the EBM-2 basal medium. Cell growth was monitored every 15 minutes for up to 7 days.

To test the effect of thrombin on proliferation of the endothelial cells, the E-plate was seeded with HUVEC cells (3 500 per well) in 150 μL of EBM-2 basal medium with ascorbic acid, hydrocortisone, heparin, gentamicin, amphotericin-B and 2% fetal bovine serum from EGM-2 SingleQuots. Subsequently, the effect of thrombin (0, 0.01, 0.05, 0.1 and 1.0 NIH U/mL) on the proliferation of HUVEC cells in the presence of VEGF_121_ standards (0, 20, 50 and 100 ng/mL) was monitored every 15 minutes for up to 7 days.

### In vitro toxicology assay kit, resazurin-based

A resazurin 4 mM stock solution was filter-sterilized and stored at -20°C. Cell proliferation was observed for up to 7 days. On day 2, 4 and 7, the cells were twice washed with PBS and incubated in 4 μM resazurin diluted in EGM-2 culture medium without added growth factors for 4 hours at 37°C and 5% CO_2_. The relative cell count was quantified by fluorescence measurement (excitation 530 nm, emission 590 nm) on a Synergy HT Multi-Mode Microplate Reader (BioTek).

### Protein concentration

The protein concentration was determined by the Bradford method [46] with bovine serum albumin (BSA) as the standard, or by the Human VEGF ELISA Kit (Invitrogen, #KHG0112), in accordance with the manufacturer’s instructions.

### Potential immune activation of cells by α_2_-PI_1-8_-VEGF_121_

The pro-inflammatory potential of α_2_-PI_1-8_-VEGF_121_ was estimated by production of selected cytokines and chemokines (Table 1) by HUVEC cells and by human monocyte-like THP-1 cells, derived from the peripheral blood of a one-year old boy with acute monocytic leukaemia (Sigma-Aldrich, Cat. No. 88081201).

**Table 1:** Cytokines and chemokines used for estimation of the cell immune activation.

Cytokine or chemokine	Abbreviation	Characteristics
Interleukin-1α	IL-1α	Protein, cytokine Produced by activated macrophages, neutrophils, epithelial cells, endothelial cells Inductor of inflammation, fever, activator of TNF-α
Interleukin-1β	IL-1β	Protein, cytokine Produced by activated macrophages Inductor of fever, inflammation, cell proliferation, differentiation, apoptosis
Granulocyte macrophage colony-stimulating factor	GM-CSF	Glycoprotein, cytokine Secreted by macrophages, T cells, mast cells, NK cells, endothelial cells and fibroblasts Stimulates production of granulocytes and monocytes
Tumor necrosis factor-α	TNF-α	Protein, cytokine Produced by macrophages, lymphoid cells, mast cells, endothelial cells, cardiac myocytes, adipose tissue, fibroblasts, neurons Inductor of inflammation, fever, apoptotic cell death, cachexia, inhibitor of tumorigenesis
Monocyte chemoattractant protein	MCP-1	Protein, chemokine Produced by endothelial, epithelial, smooth muscle, mesangial, astrocytic, monocytic and microglial cells, fibroblasts Regulates migration and infiltration of monocytes/macrophages
Interleukin-8	IL-8	Peptide, chemokine Produced by macrophages, epithelial cells, airway smooth muscle cells, endothelial cells Induces chemotaxis in granulocytes, angiogenesis, histamine production

THP-1 cells were seeded at a concentration of 10 000 cells per well in a 24-well cell culture plate (TPP, Switzerland) in the RPMI-1640 medium (Sigma-Aldrich, Cat. No. R6504) supplemented with 10% fetal bovine serum (Sigma-Aldrich, Cat. No. F7524), sodium bicarbonate (Sigma-Aldrich, Cat. No. M4892), and gentamicin (Lek, Ljubljana, Slovenia) at a concentration of 40 μg/mL. HUVEC cells, passage 5, were seeded at a density of 15 000 cells per well into 24-well cell culture plates in the modified EGM-2 medium (Lonza, Cat. No. CC-3156), supplemented with the SingleQuots^®^ Kit (Lonza, Cat. No. CC-4176) without VEGF. The reason for the slightly higher seeding density of HUVEC cells was the lower proliferation activity of these cells in comparison with THP-1 cells, as was revealed in our preliminary experiments. Recombinant α_2_-PI_1-8_-VEGF_121_ was added into both types of media at a concentration of 0 ng/mL, 20 ng/mL, and 50 ng/mL. The cells were cultured for 3 and 6 days, and then the media were collected for cytokine and chemokine analysis. The media without α_2_-PI_1-8_-VEGF_121_, taken from THP-1 or HUVEC cells, were used as a negative control.

As a positive control, lipopolysaccharides from *Escherichia coli* 026:B6 (Sigma, Cat. No. L2654) were added into the cell culture media (i.e. RPMI or EGM-2) at concentrations of 10 ng/mL, 100 ng/mL, and 1000 ng/mL on day 2 and 5. The media were collected 24 hours later for cytokine and chemokine analysis [47]. The positive controls contained no α_2_-PI_1-8_-VEGF_121_.

A Human Luminex Performance Assay Base Kit, Panel A (R&D Systems, Cat. No. LUH000) was used to analyze IL-1α, IL-1β, GM-CSF, TNF-α, MCP-1 and IL-8. The array uses color-coded microparticles, which are pre-coated with specific antibodies against cytokines or chemokines. The microparticles, incubated with samples, bind the analytes of interest. After washing, a biotinylated antibody cocktail specific to the analytes of interest is added into each well. After washing and removing the unbound antibody, streptavidin-phycoerythrin conjugate is added into each well and binds the biotinylated antibody. Finally, one laser of the Luminex analyzer determines the magnitude of the phycoerythrin signal, and the other laser determines a microparticle-specific signal of the analyte bound.

The array was processed according to the manufacturer’s protocol with some modifications (using Uniplate-Microplate Devices, 96-well, U Bottom, Whatman TM, instead of the original plate). Briefly, the samples were centrifuged at 200 g for 7 min. The microplate was pre-wetted with 100 μL of Wash Buffer, centrifuged at 900 g for 10 min. The microparticle concentrate was centrifuged at 1000 g for 30 sec, resuspended in the same solution and diluted according to the manufacturer’s protocol. The diluted microparticles (50 μL) were added into each well, followed by 50 μL of standards and media samples. Covered by an aluminium foil, the plate was incubated for 3 hours at room temperature at 500 rpm, subsequently centrifuged at 900 g for 10 min and washed 3 times with 100 μL of Wash Buffer. Then 50 μL of diluted Biotin Antibody Cocktail was added to each well. Covered by an aluminium foil, the plate was incubated for 1 hour at room temperature at 500 rpm and then washed 3 times with 100 μL of Wash Buffer. Then 50 μL of diluted Streptavidin-Phycoerythrin solution was added to each well, incubated for l hour, and subsequently the wells were washed 3 times with 100 μL of Wash Buffer. The microparticles were then resuspended in 100 μL of Wash buffer and the cytokine concentrations were analyzed on Luminex LABScan 3D (Luminex, Netherlands) from 3 parallel samples. The cytokine concentrations were normalized per 100 000 cells, similarly as in our earlier study [47].

## Results

### Expression and purification of α_2_-PI_1-8_-VEGF_121_

**Fig 1:**
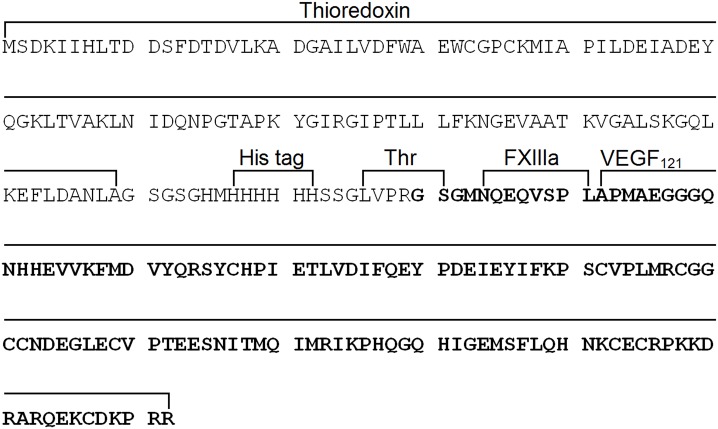
Amino acid sequence of expressed Trx–α_2_-PI_1-8_-VEGF_121_ fusion protein. His tag = hexahistidine tag; Thr = thrombin cleavage site; FXIIIa = Factor XIIIa substrate sequence NQEQVSPL; VEGF_121_ = vascular endothelial growth factor A121; resulting recombinant protein α_2_-PI_1-8_-VEGF_121_ after cleavage of the fusion part shown in bold.

**Fig 2:**
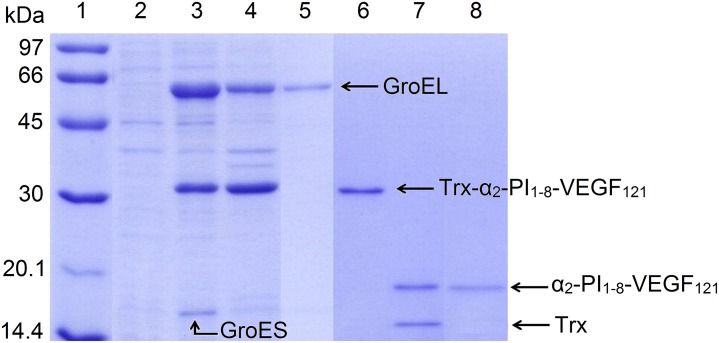
Expression in *E*. *coli* Origami B (DE3), purification and removal of the thioredoxin fusion part (Trx) of α_2_-PI_1-8_-VEGF_121_. Molecular weight markers (**1**), uninduced cells (**2**), cells induced with IPTG and L-arabinose, soluble fraction (**3**), cells induced with IPTG and L-arabinose, insoluble fraction (**4**), unbound proteins (**5**), purified fusion protein Trx-α_2_-PI_1-8_-VEGF_121_ (**6**), preparation of the fusion protein Trx-α_2_-PI_1-8_-VEGF_121_ after cleavage with thrombin (**7**), purified α_2_-PI_1-8_-VEGF_121_ (**8**).

The gene sequence encoding the modified variant of human VEGF_121_ (α_2_-PI_1-8_-VEGF_121_) was synthesized and subcloned between restriction sites *Msc* I and *Xho* I of the expression vector pET-32a(+) after the thioredoxin (Trx) gene and the hexahistidine tag. The resulting expression vector pET32-VEGF_121_ was used to transform the *E*. *coli* Origami B (DE3) strain along with plasmid pGro7 encoding bacterial chaperones GroEL/GroES. The recombinant protein Trx–α_2_-PI_1-8_-VEGF_121_ was expressed as a fusion protein composed of thioredoxin, histidine tag, thrombin cleavage site, factor XIIIa substrate sequence NQEQVSPL derived from α_2_-plasmin inhibitor and VEGF_121_ (Fig 1, S1 Text). A high level of recombinant protein expression was achieved after the induction of recombinant bacteria with 0.02 mM IPTG and subsequent culture growth at 25°C for 20–22 h. The ratio of the soluble protein fraction and the insoluble protein fraction was determined by SDS-PAGE. An analysis with Gel Analyzer software (http://www.gelanalyzer.com) showed that 43% of the Trx–α_2_-PI_1-8_-VEGF_121_ was expressed in soluble form (Fig 2, lanes 3 and 4). Trx–α_2_-PI_1-8_-VEGF_121_ was purified from the soluble fraction of cytoplasmic proteins using Talon Metal Affinity Resin (Fig 2, lane 6). After purification, the fusion partner was cleaved out with thrombin. The molecular mass of the Trx–α_2_-PI_1-8_-VEGF_121_ fusion protein estimated by SDS-PAGE was 32.5 kDa (theoretical molecular mass 29.2 kDa). Cleavage of the fusion partner containing the hexahistidine tag with thrombin yielded a thioredoxin fragment (15.1 kDa, theoretical molecular mass 13.9 kDa) and α_2_-PI_1-8_-VEGF_121_ (18.8 kDa, theoretical molecular mass 15.3 kDa; Fig 2, line 7). The yield of purified α_2_-PI_1-8_-VEGF_121_ determined by the Bradford protein assay was 1.4 mg protein per 1 L of culture medium.

### Mitogenic activity of α_2_-PI_1-8_-VEGF_121_

To test the mitogenic activity of α_2_-PI_1-8_-VEGF_121_, the proliferative effect on HUVEC cells was investigated using the xCELLigence system and a resazurin-based assay. The activity of recombinant α_2_-PI_1-8_-VEGF_121_ was compared to the activity of VEGF_121_ from commercial sources. For this purpose, we used a VEGF_121_ variant expressed in *E*. *coli* and a variant expressed in mammalian HEK cells. Three concentrations of VEGF_121_ (20, 50 and 100 ng/mL) were used for the evaluation. The concentrations of each VEGF_121_ variant were determined by ELISA analysis. The EGM-2 cultivation medium without growth factors served as a negative control.

**Fig 3:**
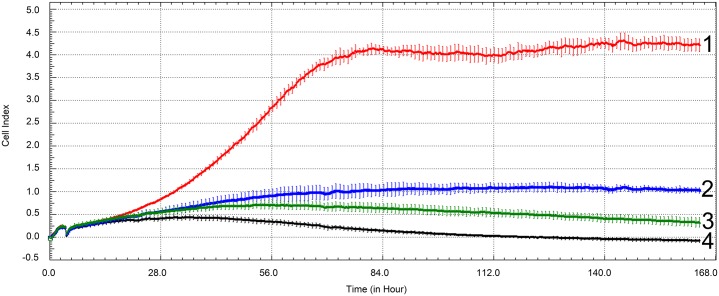
VEGF mitogenic activity evaluation using the xCELLigence system. The mitogenic activity of α_2_-PI_1-8_-VEGF_121_ and commercial VEGF_121_ standards was evaluated using real-time monitoring of HUVEC cell proliferation. α_2_-PI_1-8_-VEGF_121_, 50 ng/mL (**1**), VEGF_121_ **I**, 50 ng/mL (**2**), VEGF_121_ **II**, 50 ng/mL (**3**), negative control (culture medium without VEGF) (**4**). The cells were incubated for 165 hours. The results shown here are mean ± SEM (n = 4).

The evaluation showed that the recombinant α_2_-PI_1-8_-VEGF_121_ manufactured in this work had a greater impact on HUVEC proliferation than the two tested commercially-available VEGF_121_ variants. This effect was most apparent at a concentration of 50 ng/mL of VEGF_121_ (see Fig 3, S3 Fig), but it was also observed at VEGF_121_ concentrations of 20 and 100 ng/mL (see S1 and S7 Figs).

The plateau value of the cell index (evaluated at 165 hours, i.e. on day 7) for α_2_-PI_1-8_-VEGF_121_ was 4.30 ± 0.20; for VEGF_121_ **I** it was 1.11 ± 0.11; for VEGF_121_ **II** it was 0.72 ± 0.10, and for the culture medium without VEGF_121_ the value was 0.43 ± 0.06 (Fig 3). In other words, PI_1-8_-VEGF_121_ expressed and purified under the described conditions showed an approximately 3.9 times greater effect on endothelial growth in comparison with the mitogenic activity of the VEGF_121_ **I** standard, and an approximately 6.0 times greater effect when compared to the mitogenic activity of VEGF_121_ **II** (Fig 3).

**Fig 4:**
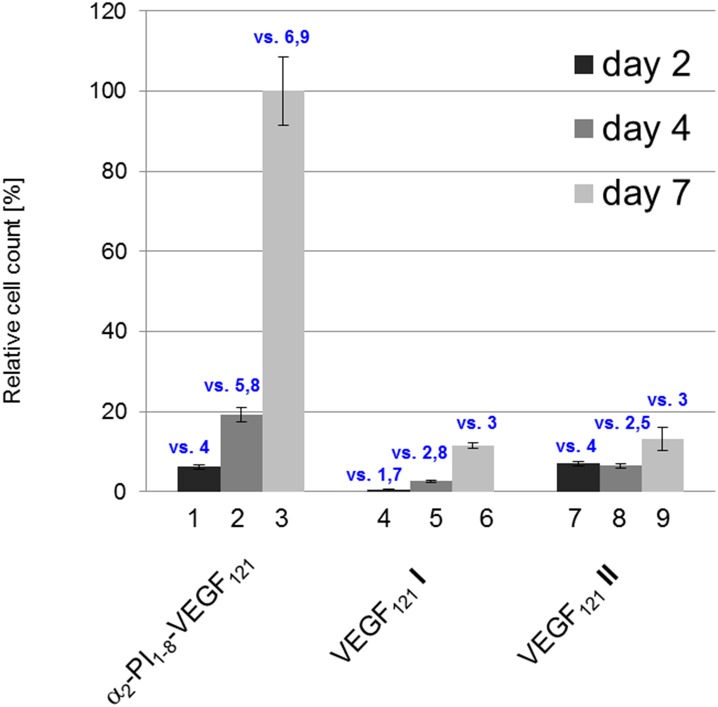
The comparison of endothelial cell proliferation in media containing VEGF_121_ (50 ng/ mL) from different sources. Relative cell count in a cultivation medium containing the examined VEGF_121_ preparations (α_2_-PI_1-8_-VEGF_121_ from this work, VEGF_121_ **I** expressed in *E*. *coli*, and VEGF_121_ **II** expressed in HEK cells) assessed with the fluorescent indicator resazurin after 48 hours (day 2), 96 hours (day 4), and 165 hours (day 7) of incubation, compared to the control with no VEGF (= 0%). The VEGF concentration in all preparations was 50 ng/mL. Results shown as mean ± SEM (n = 4). The statistical significance was determined by the ANOVA, Student–Newman–Keuls method; p<0.05 in comparison with the samples indicated by numbers (in blue) above the columns.

The data obtained in the xCELLigence system were further supported by an independent proliferation assay based on the fluorescent indicator resazurin, performed on the 2^nd^, 4^th^ and 7^th^ day of cultivation. Major differences were observed after 165 hours (day 7) of incubation in a cultivation medium containing 50 ng/mL of VEGF_121_ preparations, where the relative cell counts were 11.5 ± 0.6% for VEGF_121_ **I** and 13.3 ± 2.7% for VEGF121 **II**, in comparison with recombinant α_2_-PI_1-8_-VEGF_121_ (100%, Fig 4). This effect was also observed at VEGF_121_ concentrations of 20 and 100 ng/mL (see S2 and S4 Figs). Thus, α_2_-PI_1-8_-VEGF_121_ increased endothelial proliferation 8.7 times in comparison with VEGF_121_ **I** and 7.5 times in comparison with VEGF_121_ **II** (Fig 4).

### Effect of thrombin on HUVEC proliferation

**Fig 5:**
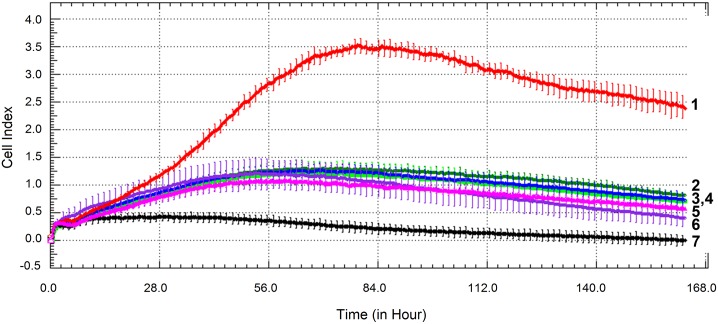
The effect of thrombin (0.01, 0.05, 0.1 and 1.0 NIH U/mL) on the proliferation of HUVEC cells. Seeding density 3 500 cells per well of a 96-well E-plate in EBM-2 basal medium supplemented with ascorbic acid, hydrocortisone, heparin, gentamicin, amphotericin-B and 2% fetal bovine serum from EGM-2 SingleQuots and in the presence of the commercial VEGF_121_ **I** standard (50 ng/mL) or α_2_-PI_1-8_-VEGF_121_ followed by the xCELLigence system.

As revealed by the real-time monitoring of cell growth using the xCELLigence system, the addition of thrombin in concentrations of 0.01, 0.05, 0.1 and 1.0 NIH U/mL to the media with VEGF_121_ standards (concentrations of 20, 50 and 100 ng/mL) did not significantly change the proliferation activity of HUVEC cells. The growth dynamics of HUVEC in the presence of the thrombin + VEGF_121_ standards were similar as in the presence of the VEGF_121_ standards only (Fig 5, S5 Fig).

Curves: α_2_-PI_1-8_-VEGF_121_ (**1**), control VEGF_121_ **I** (**2**), control VEGF_121_ **I** + thrombin 0.01 NIH U/mL (**3**), control VEGF_121_ **I** + thrombin 0.05 NIH U/mL (**4**), control VEGF_121_ **I** + thrombin 0.10 NIH U/mL (**5**), control VEGF_121_ **I** + thrombin 1.00 NIH U/mL (**6**), negative control: no VEGF_121_ + no thrombin (**7**).

The mitogenic activity of the preparations (controls) was evaluated using real-time monitoring of HUVEC cell proliferation. The cells were monitored every 15 minutes for 163 hours. The results shown here are mean ± SEM (n = 4). VEGF_121_ **I** expressed in *E*. *coli* (50 ng/mL) was used as a standard.

### Cell immune activation by VEGF_121_

**Fig 6:**
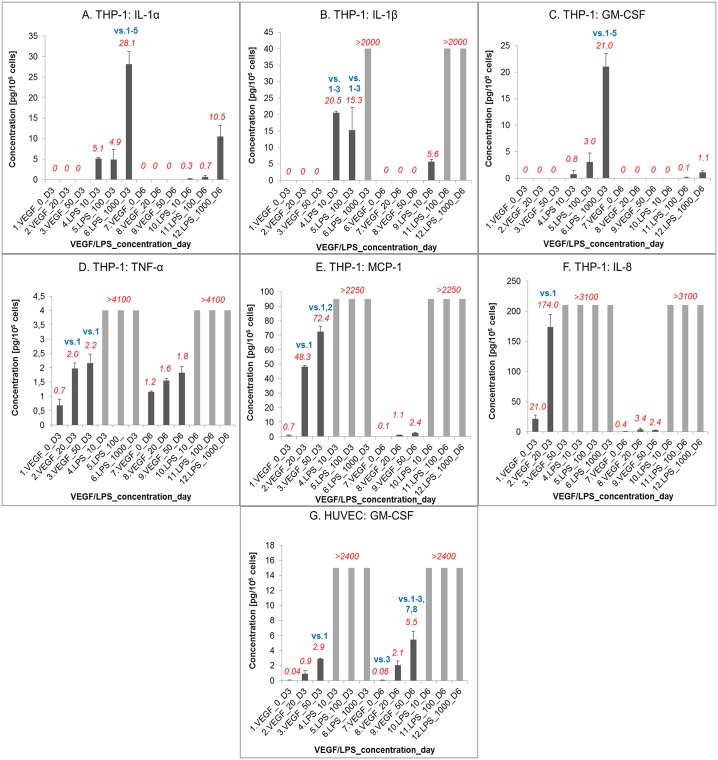
Cell immune activation by VEGF_121_ determined by Luminex Performance Assay. Concentration of IL-1α (A), IL-1ß (B), GM-CSF(C, G), TNF-α (D), MCP-1 (E) and IL-8 (F) produced by THP-1 cells (A-F) or HUVEC (G) in the cell culture medium on day 3 (D3) or day 6 (D6) of cultivation in media with 0, 20 and 50 ng/mL of α_2_-PI_1-8_-VEGF_121_ (VEGF) or 10, 100 or 1000 ng/mL of bacterial lipopolysaccharide (LPS). The cells were seeded into 24-well culture plates in 1.5 ml of the media (THP-1: 10 000 cells/well, RPMI-1640 medium; HUVEC: 15 000 cells/well, EGM-2 medium without commercial VEGF_121_ supplement). The amount of cytokines or chemokines in the media was calculated per 10^5^ cells. Mean ± S.E.M. (standard error of mean) from 3 measurements for each experimental group and time interval. The mean value is shown in italics (in red) above the columns. Values higher than the calibration curve are indicated with “>”. The statistical significance was determined by the ANOVA, Student–Newman–Keuls method; p<0.05 in comparison with the samples indicated by numbers (in blue) above the columns.

The Luminex Performance Assay revealed that THP-1 monocyte-like cells, cultured with α_2_-PI_1-8_-VEGF_121_ (in a concentration of 0, 20 or 50 ng/mL), did not produce Il-1α, Il-1ß and GM-CSF cytokines in a concentration detectable in the cell culture media. However, when the THP-1 cells were stimulated with bacterial lipopolysaccharide (LPS, concentrations from 10 to 1000 ng/mL), i.e. an endotoxin often used as a positive control in studies of cell immune activation, these cells produced Il-1α, Il-1ß, and GM-CSF in high concentrations (Fig 6A–6C, S6 Fig). However, α_2_-PI_1-8_-VEGF_121_ stimulated the THP-1 cells to produce TNF-α, MCP-1 and IL-8. The production of these molecules was proportional to the α_2_-PI_1-8_-VEGF_121_ concentration, but it was rather transient and was apparent in considerable amounts only for 3 days. After 6 days of culture, the concentration of TNF-α, MCP-1 and IL-8 in the cell culture media dropped to very low values (Fig 6D–6F, S6 Fig). However, LPS massively stimulated the production of TNF-α, MCP-1 and IL-8 by THP-1 cells at both time intervals, so their concentrations in the media exceeded the standard calibration curve of the array, even at the lowest LPS concentration of 10 ng/mL. As for the HUVEC cells, α_2_-PI_1-8_-VEGF_121_ stimulated the production of GM-CSF, but this production was markedly lower that the values obtained after stimulation with LPS, even in the lowest concentration (Fig 6G, S6 Fig). Thus, the potential of our recombinant VEGF_121_ to induce cell immune activation and an inflammatory reaction can be qualified as relatively low.

## Discussion

Most studies on VEGF expression in bacteria describe its purification from insoluble inclusion bodies by the denaturing and refolding method, where the recovery of protein biological activity may be a problem [30, 48–53]. However, only a small number of studies have shown the heterologous expression of VEGF in *E*. *coli* in the soluble protein fraction [38, 54]. Our work is one of the rare cases in which VEGF is expressed in *E*. *coli* in a soluble form.

VEGF belongs to the cystine knot superfamily of growth factors. The cystine knot consists of nine cysteine residues that are present within the VEGF_121_ structure and play a role in protein dimerization. Due to the absence of a hydrophobic core region, the cystine knot is believed to be the major determinant of protein stability [55]. This complex structure may be responsible for the higher requirements on protein folding quality when expressed heterologously. In accordance with studies that have also dealt with the expression of proteins with a similar structure [38–40], pET-32a(+) vector and *E*. *coli* Origami B (DE3) host cells were chosen for α_2_-PI_1-8_-VEGF_121_ production. Moreover, to ameliorate protein folding, α_2_-PI_1-8_-VEGF_121_ was co-expressed with GroEL/ES chaperones, which have been reported to increase the solubility, the yield, and in some cases even the biological activity of several recombinant proteins [56–62]. To the best of our knowledge, co-expression of VEGF protein with molecular chaperones has not been published before.

About 43% of recombinant thioredoxin-α_2_-PI_1-8_-VEGF_121_ fusion protein was expressed in the soluble protein fraction. The corresponding molecular mass was found to be 32.5 kDa (Fig 2), which was in a good agreement with the expected theoretical size of the fusion protein (29.2 kDa). Two other dominant protein bands present in the soluble protein fraction on an SDS-PAGE gel correspond to a molecular mass of ca 58 and 16 kDa (Fig 2, column l and 3). These protein bands were interpreted as molecular chaperones GroEL and GroES. The putative GroEL protein was present in both the soluble protein fraction and the insoluble protein fraction (Fig 2, lanes 3, 4). The apparent presence in a fraction of unbound proteins suggests a weak interaction with Talon Metal Affinity Resin, which was used for affinity chromatography (Fig 2, lane 5).

During dialysis following affinity chromatography, an unspecified amount of the protein precipitated. Thus, although the expression of the soluble protein fraction was high (Fig 2), the overall yield of 1.4 mg per L of culture was lower than expected. The yield of recombinantly prepared VEGF mentioned in related studies varies on a case-by-case basis. Some studies do not present any yield [49, 54] or the data are unclear [38]. In other works, the yield of purified active VEGF_121_ or VEGF_165_ is between 1 mg and 5 mg per L of bacterial culture [50, 52, 53], which is similar to the yield of mutant VEGF_121_ purified in this work. The yield of recombinantly expressed α_2_-PI_1-8_-VEGF_121_ by Zisch et al. was 11 mg/L [30].

The SDS-PAGE record of purified α_2_-PI_1-8_-VEGF_121_ did not show any contamination, but it is possible that small traces of thrombin used for cleavage of the thioredoxin fusion part remained in the solution. It has been reported that a low concentration of thrombin is able to stimulate the proliferation of endothelial cells. In earlier studies, the addition of thrombin to HUVEC cells cultivated in serum-free conditions resulted in significant dose- and time-dependent stimulation of endothelial cell proliferation, with a maximal effect at concentrations of thrombin of ca 0.04–0.08 NIH U/mL [41–43]. However, in our study, similar or even higher concentrations of thrombin (0.01, 0.05, 0.1 and 1.0 NIH U/mL) had no significant influence on the HUVEC proliferation (Fig 5). A positive effect of thrombin on the very high mitogenic activity of α_2_-PI_1-8_-VEGF_121_ can therefore be excluded.

After cleavage of the thioredoxin fusion part by thrombin, the mitogenic activity of α_2_-PI_1-8_-VEGF_121_ was evaluated by two independent methods, namely by real-time monitoring of endothelial cell proliferation by the xCELLigence system, and by the fluorescent indicator resazurin. Both assays revealed that α_2_-PI_1-8_-VEGF_121_ expressed and purified under the conditions described here showed a significantly greater effect on endothelial cell growth than the mitogenic activity of the VEGF_121_ **I** and **II** standards. This effect was the most apparent after 7 days, when, at a concentration of 50 ng/mL, α_2_-PI_1-8_-VEGF_121_ increased the endothelial cell proliferation approximately 3.9 to 8.7 times more than the VEGF_121_ **I** and II (Figs 3 and 4). The concentrations of all VEGF_121_ preparations used in this work were determined by VEGF-ELISA, under the same experimental conditions (see S1 Table). This suggests that the relative VEGF_121_ activity was not influenced by incorrect determination of the VEGF_121_ concentration. The highly mitogenic α_2_-PI_1-8_-VEGF_121_ created in this study (even with a relatively low yield) could be used effectively for improving of endothelialization of various biomaterials, where it could be incorporated in lower concentrations than those usually applied. This option would be helpful for reducing undesirable side-effects of VEGF, such as inflammation, induced by overexpression of VEGF [45].

Recombinant α_2_-PI_1-8_-VEGF_121_ contains an additional N-terminal substrate sequence (NQEQVSPL) for factor XIIIa, but there is no evidence that such a short peptide should influence the biological activity of VEGF_121_. Studies comparing the biological activities of various VEGF preparations have not found any significant differences [53, 63]. The biological activity was also comparable to that of the commercial standard in the case of VEGF proteins joined to uncleaved fusion partners, such as thioredoxin or GST [49, 50].

Work evaluating the biological activity of recombinant VEGF with an uncleaved C-terminal GST fusion partner shows that the presence of GST did not affect the correct assembly of dimers and the display of residues critical for receptor recognition [50]. A similar finding was reported in a study describing the functionality of VEGF_165_ N-terminally fused to thioredoxin, where the authors found that N-terminal extension decreased the affinity of VEGF fusion proteins to VEGFR-2, but at saturated concentrations these proteins were as efficient as VEGF_165_ of the correct size [49]. Unlike the activity of our α_2_-PI_1-8_-VEGF_121_, which was 8.7 times higher than the activity of the VEGF_121_ **I** standard, the activity of α_2_-PI_1-8_-VEGF_121_ prepared without chaperones [30] was only comparable with the activity of unmodified, *E*. *coli*-derived VEGF_121_. However, in many papers there is no comparison of biological activity with a commercial VEGF standard [38, 48, 51, 52, 54].

It has been shown that administering the thioredoxin gene can stimulate the proliferation of mesenchymal stem cells [64]. When thioredoxin is added into the media in the form of a protein, concentrations as high as 5–70 μg/mL were observed to have an impact on the cell growth [65]. Regarding the level of efficient thioredoxin concentration, the high mitogenic activity of the manufactured VEGF observed in our experiments should not be related to the traces of hypothetical thioredoxin contamination, which were far below 50 ng/mL. The his-tagged *E*. *coli* thioredoxin, which was used in this work as a fusion partner, was cleaved out and removed by affinity chromatography. Its elimination was confirmed by SDS-PAGE analysis of the final VEGF_121_ preparation. In addition, as mentioned above, the biological activity of recombinant VEGF was not increased even if its fusion partner, thioredoxin, was not cleaved out [49, 50].

Thus, a positive role of thrombin, NQEQVSPL or thioredoxin in the increased mitogenic activity of our recombinant α_2_-PI_1-8_-VEGF_121_ can be ruled out. However, it should be noted that the commercial VEGF_121_ standards that were available to us (i.e., purchased from Prospecbio, Cat. No. CYT-343 and CYT-116) were obtained in the form of a lyophilized powder recommended to be reconstituted in PBS. Proteins in general may lose their biological activity during lyophilization or reconstitution. Presumably, all this could reduce the biological activity of the VEGF_121_ standards.

The high mitogenic activity α_2_-PI_1-8_-VEGF_121_ created in this study may be caused by co-expression of the growth factor with molecular chaperones that have not been used so far in VEGF production. A similar phenomenon was observed in the case of several recombinant proteins expressed in *E*. *coli*. For example, the relative binding activity of anti-B-type natriuretic peptide scFv [57], and the specific enzyme activities of nitrilase [56], and also cold-active lipase Lip-948 [66], were increased by co-expression with molecular chaperones. In the case of VEGF, chaperones could either stabilize an optimal folding structure during the expression, or could selectively solubilize a highly active VEGF fraction. However, this interesting but complicated issue needs further investigation.

Our analysis of cell immune activation suggested that the potential of α_2_-PI_1-8_-VEGF_121_ to induce the production of inflammatory cytokines and chemokines in cells and their release into the cell culture media is relatively low. This conclusion is based on our findings that upon stimulation by α_2_-PI_1-8_-VEGF_121_, human monocyte-like THP-1 cells did not release measurable quantities of IL-1α, IL-1β and GM-CSF, and released only small amounts of IL-8, TNF- α and MCP-1 in comparison to cells stimulated by LPS. HUVEC cells were able to release only GM-CSF, but its concentration was again very low compared with the values obtained after LPS stimulation. These results can be considered as favorable for our intended future use of α_2_-PI_1-8_-VEGF_121_ for incorporation into fibrin matrices for potential modification of cardiovascular implants and scaffolds for tissue engineering in order to improve their endothelialization or vascularization. However, in other studies, both recombinant and natural VEFG molecules have been reported to act as pro-inflammatory factors, which activated cells of the immune system (leucocytes, lymphocytes, monocytes and macrophages), and also vascular endothelial cells to produce pro-inflammatory cytokines, chemokines, and adhesion molecules of immunoglobulin and selectin families [44, 45]. In addition, recombinant VEGF molecules are capable of inducing the production of antibodies when administered into organisms *in vivo*. This has been used for producing vaccines against tumors [67, 68]. A vaccine based on human recombinant VEGF combined with a bacterial adjuvant has even been tested in a phase I clinical trial on patients with advanced solid tumors [69].

## Conclusion

In this work we have described a new procedure by which a highly active mutant variant α_2_-PI_1-8_-VEGF_121_ can be produced. The mutant protein structure was first designed by Zisch et al. [30], and was used for preparing fibrin gels with incorporated VEGF_121_. The new procedure, based on co-expressing thioredoxin-α_2_-PI_1-8_-VEGF_121_ with recombinant molecular chaperones GroES/EL, resulted in mitogenic activity of α_2_-PI_1-8_-VEGF_121_ that was 3.9–8.7 times higher than the mitogenic activity of commercial VEGF_121_ standards. Very high mitogenic activity and a low effect on inducing the inflammatory activation of human endothelial HUVEC cells and human monocyte-like THP-1 cells make this α_2_-PI_1-8_-VEGF_121_ variant attractive for fibrin-based biomaterials releasing VEGF_121_, e.g. for coating cardiovascular implants in order to improve their endothelialization.

## Supporting Information

S1 Dataset SDS-PAGE outputs of expression in *E*. *coli* Origami B (DE3), purification and removal of the thioredoxin fusion part (Trx) of α_2_-PI_1-8_-VEGF_121_. (DOCX)

S1 Fig Mitogenic activity of VEGF_121_ (20, 50 and 100 ng/mL) evaluated using the xCELLigence system. The mitogenic activity of the α_2_-PI_1-8_-VEGF_121_ and commercial VEGF_121_ standards was evaluated using real-time monitoring of HUVEC cell proliferation. Curves: α_2_-PI_1-8_-VEGF_121_, 20, 50 and 100 ng/mL (**3, 2, 1**), VEGF_121_ **I**, 20, 50 and 100 ng/mL (**5, 4, 6**), VEGF_121_ **II**, 20, 50 and 100 ng/mL (**9, 8, 7**), negative control (culture medium without VEGF) (**10**). The cells were incubated for 165 hours. The results shown here are mean ± SEM (n = 4).
(TIF)

S2 Fig The comparison of endothelial cell proliferation in media containing VEGF_121_ (20 and 100 ng/ mL) from different sources. Relative cell count in a cultivation medium containing the examined VEGF_121_ preparations (α_2_-PI_1-8_-VEGF_121_ from this work, VEGF_121_ **I** expressed in *E*. *coli*, and VEGF_121_ **II** expressed in HEK cells) assessed with the fluorescent indicator resazurin after 48 hours (day 2), 96 hours (day 4) and 165 hours (day 7) of incubation, compared to the control with no VEGF (= 0%). The VEGF_121_ concentration in all preparations was 20 ng/mL (A) and 100 ng/mL (B). Results shown as mean ± SEM (n = 4). The statistical significance was determined by the ANOVA, Student–Newman–Keuls method; p<0.05 in comparison with the samples indicated by numbers (in blue) above the columns.
(TIF)

S3 Fig xCELLigence system output (VEGF mitogenic activity evaluation). The mitogenic activity of α_2_-PI_1-8_-VEGF_121_ and commercial VEGF_121_ standards evaluated using real-time monitoring of HUVEC cell proliferation. α_2_-PI_1-8_-VEGF_121_, 50 ng/mL (**1**), VEGF_121_ **I**, 50 ng/mL (**2**), VEGF_121_ **II**, 50 ng/mL (**3**), negative control (culture medium without VEGF) (**4**). Excel sheet of raw data (mean ± standard error of the mean (S.E.M.)).
(XLSX)

S4 Fig Microplate reader fluorescence measurement output (proliferation assay based on the fluorescent indicator resazurin for VEGF concentration 20, 50 and 100 ng/mL). Relative cell count in a cultivation medium containing the examined VEGF_121_ preparations (α_2_-PI_1-8_-VEGF_121_ from this work, VEGF_121_ **I** expressed in *E*. *coli*, and VEGF_121_ **II** expressed in HEK cells) assessed with the fluorescent indicator resazurin after 48 hours (day 2), 96 hours (day 4), and 165 hours (day 7) of incubation, compared to the control with no VEGF (= 0%). The VEGF concentration in preparations was 20, 50 and 100 ng/mL. Excel sheet of raw data (mean ± S.E.M.).
(XLSX)

S5 Fig xCELLigence system output (the effect of thrombin (0.01, 0.05, 0.1 and 1.0 NIH U/mL) on the proliferation of HUVEC cells). α_2_-PI_1-8_-VEGF_121_ (**1**), control VEGF_121_ **I** (**2**), control VEGF_121_ **I** + thrombin 0.01 NIH U/mL (**3**), control VEGF_121_ **I** + thrombin 0.05 NIH U/mL (**4**), control VEGF_121_ **I** + thrombin 0.10 NIH U/mL (**5**), control VEGF_121_ **I** + thrombin 1.00 NIH U/mL (**6**), negative control: no VEGF + no thrombin (**7**). Excel sheet of raw data (mean ± S.E.M.).
(XLSX)

S6 Fig Cell immune activation by VEGF determined by Luminex Performance Assay. Concentration of IL-1α (A), IL-1ß (B), GM-CSF(C, G), TNF-α (D), MCP-1 (E) and IL-8 (F) produced by THP-1 cells (A-F) or HUVEC (G) in the cell culture medium on day 3 (D3) or day 6 (D6) of cultivation in media with 0, 20 and 50 ng/mL of α_2_-PI_1-8_-VEGF_121_ (VEGF) or 10, 100 or 1000 ng/mL of bacterial lipopolysaccharide (LPS). Excel sheet of raw data from 3 measurements.
(XLSX)

S7 Fig xCELLigence system output (VEGF mitogenic activity evaluation; VEGF 20, 50 and 100 ng/mL). α_2_-PI_1-8_-VEGF_121_, 20, 50 and 100 ng/mL (**3, 2, 1**), VEGF_121_ **I**, 20, 50 and 100 ng/mL (**5, 4, 6**), VEGF_121_ **II**, 20, 50 and 100 ng/mL (**9, 8, 7**), negative control (culture medium without VEGF) (**10**). Excel sheet of raw data (mean ± S.E.M.).
(XLSX)

S1 Table VEGF concentration determination. Due to the risk of underestimating the potential VEGF_121_ concentration, we determined the VEGF_121_ protein concentration independently. The VEGF_121_ solutions were analyzed simultaneously by the FluoroProfile Protein quantification Kit and by VEGF-ELISA, in order to confirm whether VEGF-ELISA can detect VEGF in different solutions with same sensitivity. Our results suggest that VEGF-ELISA detected the VEGF in all VEGF solutions with similar sensitivity, and provided results that are 1.07–1.70 times higher than those determined by FluoroProfile Protein quantification Kit.
(DOCX)

S2 Table VEGF concentration determination using FluoroProfile Protein Quantification Kit (Cat. No. FP0010, Sigma-Aldrich) for protein concentration determination. Excel sheet of raw data.
(XLSX)

S3 Table VEGF concentration determination VEGF121-ELISA Kit (Cat. No. KHG0112, Invitrogen). Excel sheet of raw data.
(XLSX)

S1 Text Nucleotide sequence of α_2_-PI_1-8_-VEGF_121_ gene and amino acid sequence of expressed Trx–α_2_-PI_1-8_-VEGF_121_ fusion protein. (DOC)
