# Correction: Enhanced Mitogenic Activity of Recombinant Human Vascular Endothelial Growth Factor VEGF_121_ Expressed in *E*. *coli* Origami B (DE3) with Molecular Chaperones

**DOI:** 10.1371/journal.pone.0169590

**Published:** 2017-01-03

**Authors:** Ondřej Kaplan, Jana Zárubová, Barbora Mikulová, Elena Filová, Jiřina Bártová, Lucie Bačáková, Eduard Brynda

There are errors in the author affiliations. The affiliations should appear as shown here:

Ondřej Kaplan^1,2^, Jana Zárubová^2^, Barbora Mikulová^2,3,4^, Elena Filová^2^, Jiřina Bártová^5^, Lucie Bačáková^2^, Eduard Brynda^1^

**1** Institute of Macromolecular Chemistry, Czech Academy of Sciences, CZ-162 06, Prague, Czech Republic, **2** Institute of Physiology, Czech Academy of Sciences, CZ-142 20, Prague, Czech Republic, **3** Institute of Microbiology, Czech Academy of Sciences, CZ-142 20, Prague, Czech Republic, **4** Faculty of Science, Charles University in Prague, CZ-128 40, Prague, Czech Republic, **5** School of Dental Medicine, General University Hospital in Prague, CZ-128 08, Prague, Czech Republic
