# Supplementary figures and images for: Enhanced Mitogenic Activity of Recombinant Human Vascular Endothelial Growth Factor VEGF121 Expressed in E. coli Origami B (DE3) with Molecular Chaperones
Source: PLoS One. 2016 Oct 7;11(10):e0163697. doi: 10.1371/journal.pone.0163697 (PMC5055331; doi:10.1371/journal.pone.0163697)

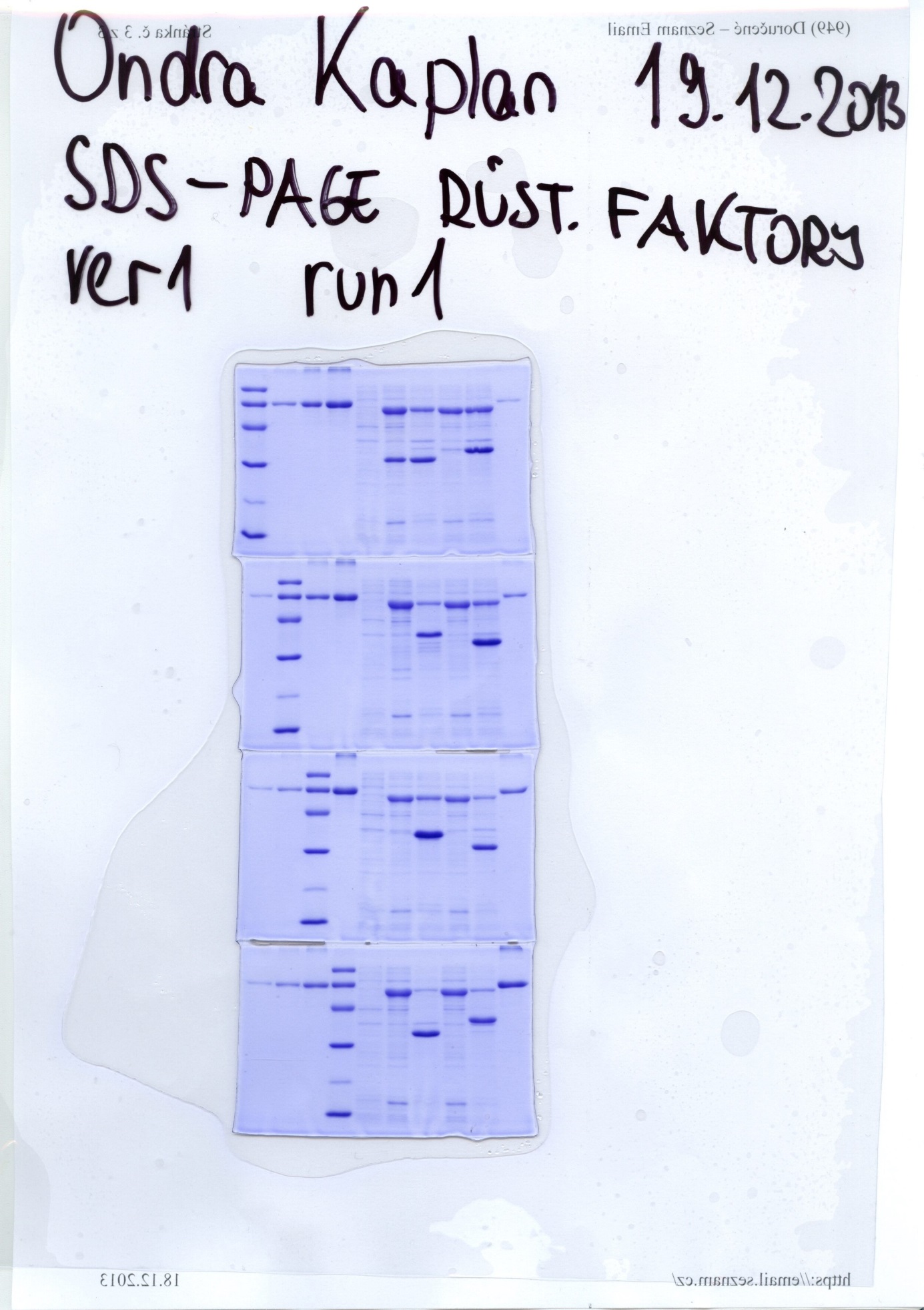


SDS_PAGE_20_01_2014_Run1_ver2_Purif


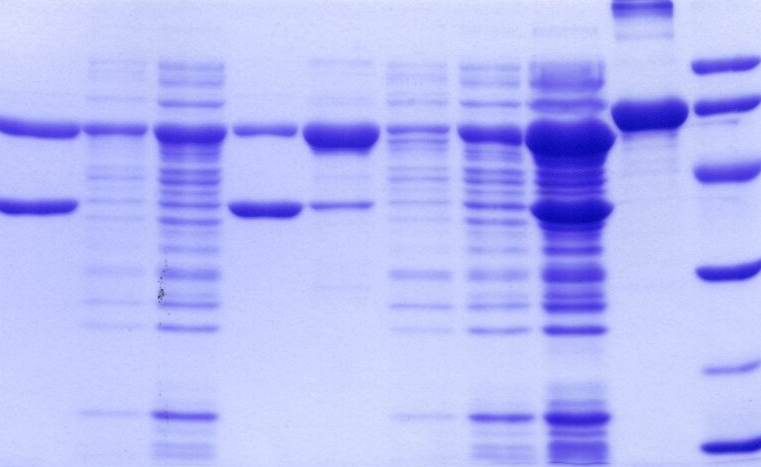


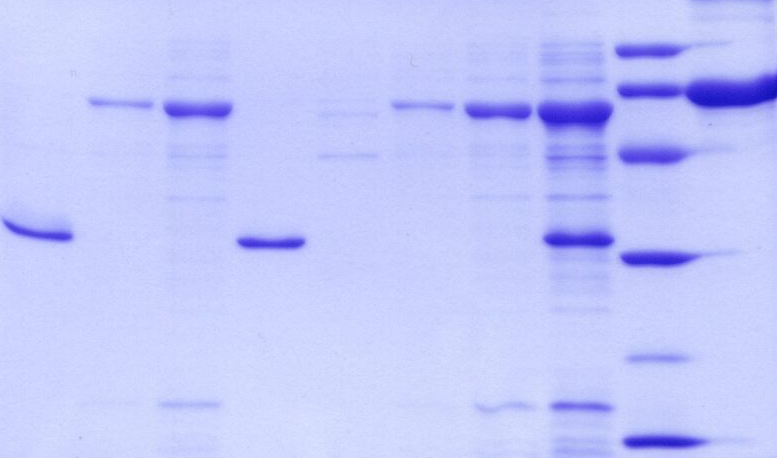


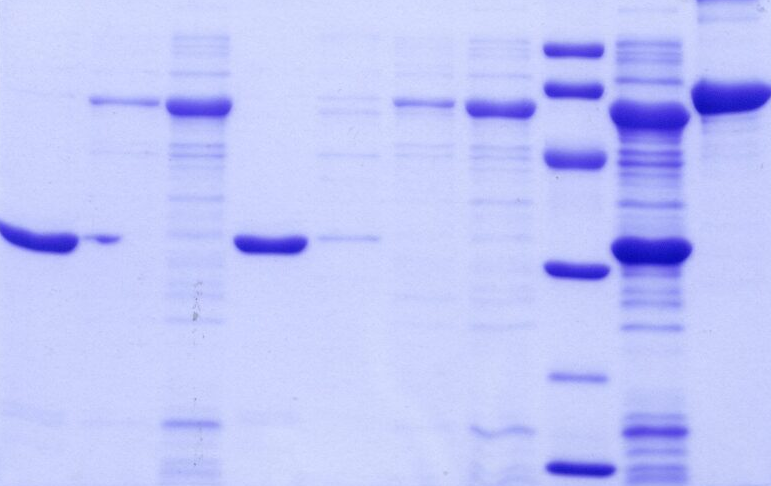


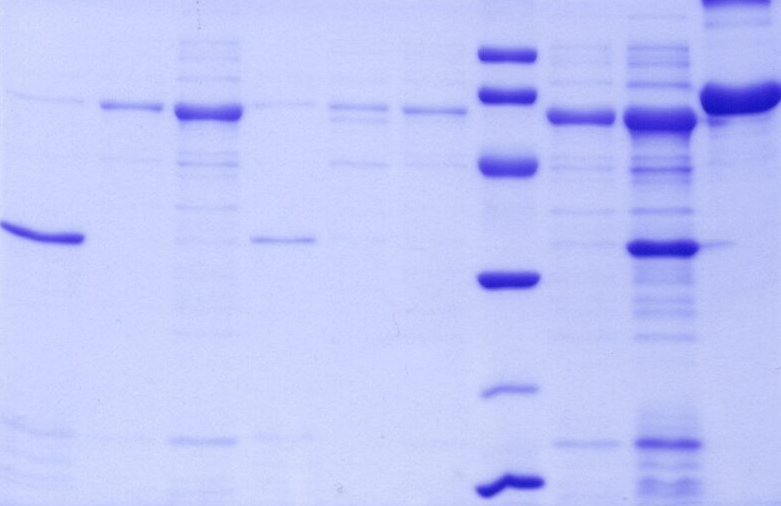


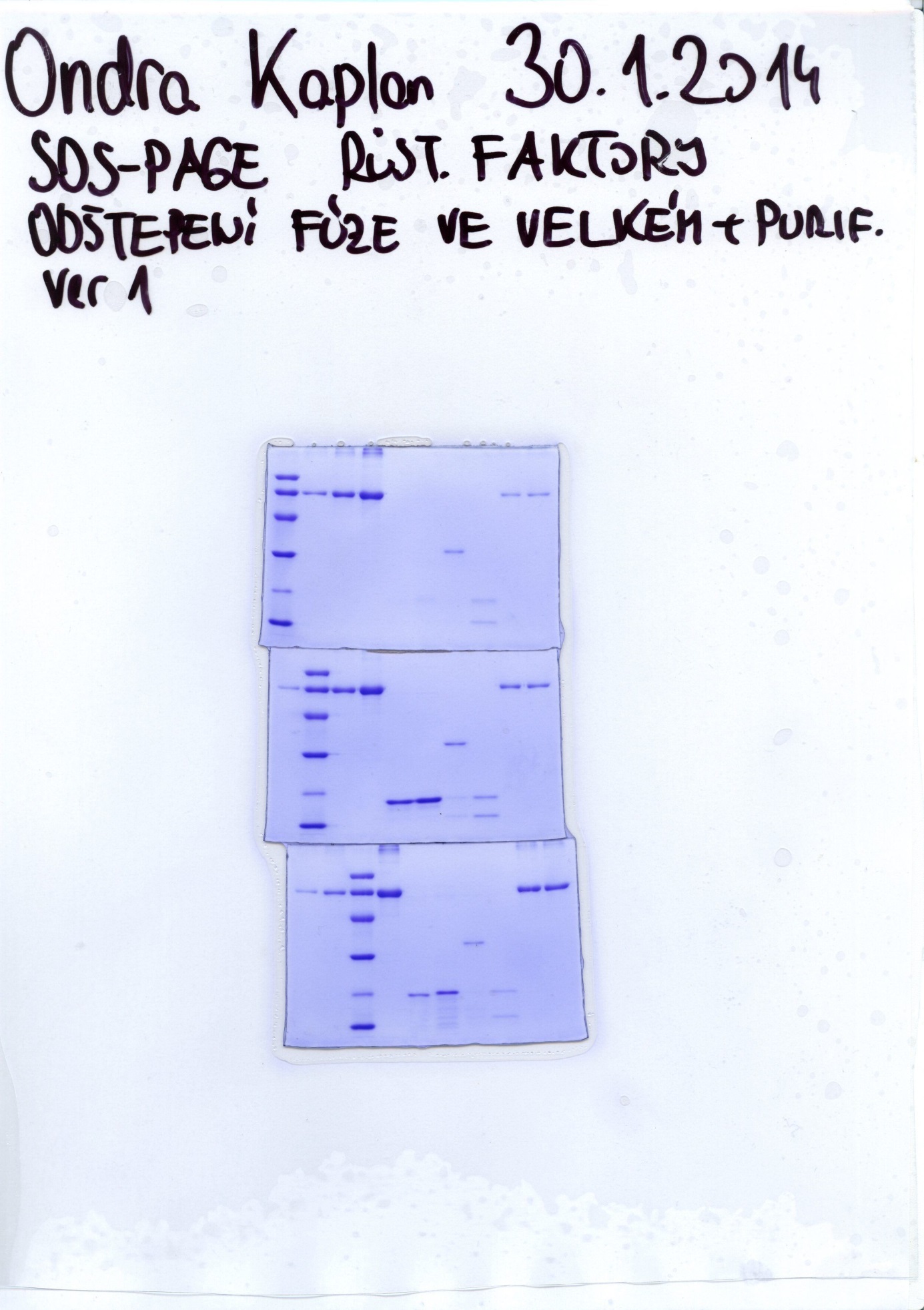

Supplement: S1 Dataset — (DOCX) [file pone.0163697.s001.docx]

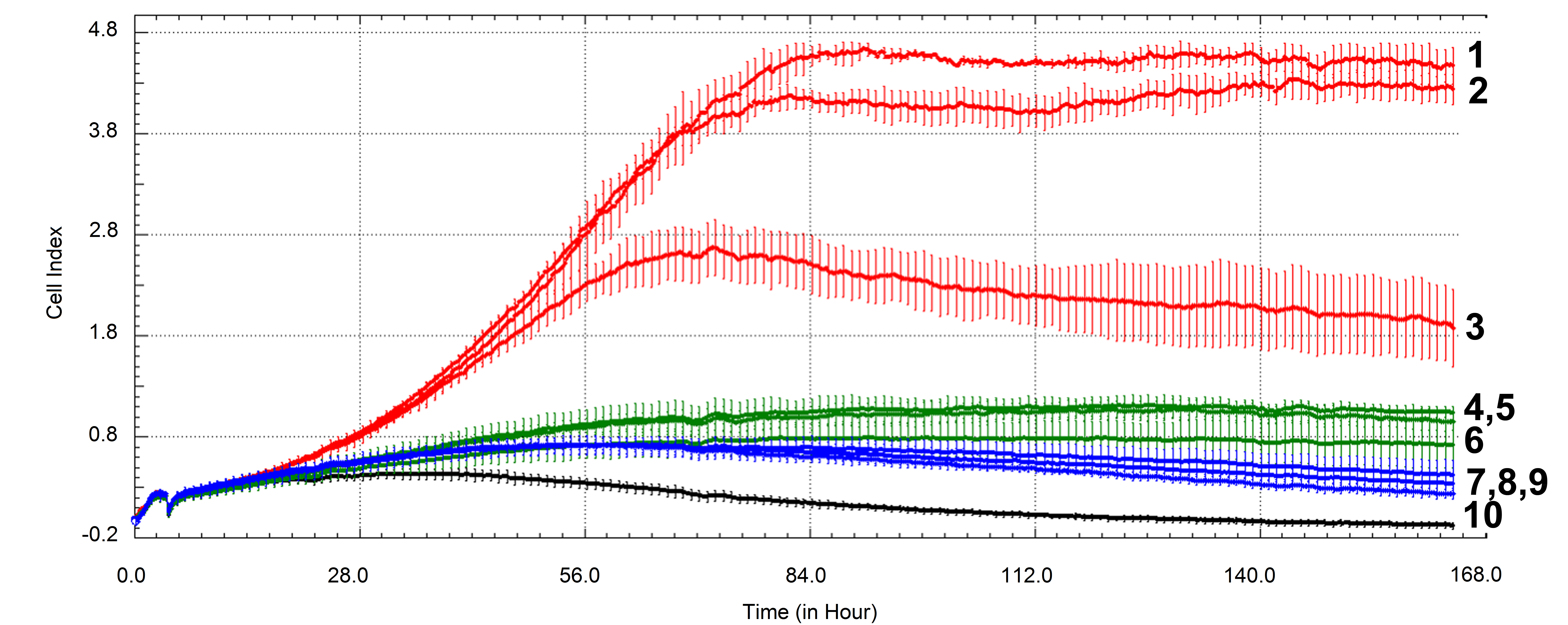

Supplement: S1 Fig — The mitogenic activity of the α2-PI1-8-VEGF121 and commercial VEGF121 standards was evaluated using real-time monitoring of HUVEC cell proliferation. Curves: α2-PI1-8-VEGF121, 20, 50 and 100 ng/mL (3, 2, 1), VEGF121 I, 20, 50 and 100 ng/mL (5, 4, 6), VEGF121 II, 20, 50 and 100 ng/mL (9, 8, 7), negative control (culture medium without VEGF) (10). The cells were incubated for 165 hours. The results shown here are mean ± SEM (n = 4). (TIF) [file pone.0163697.s002.tif]

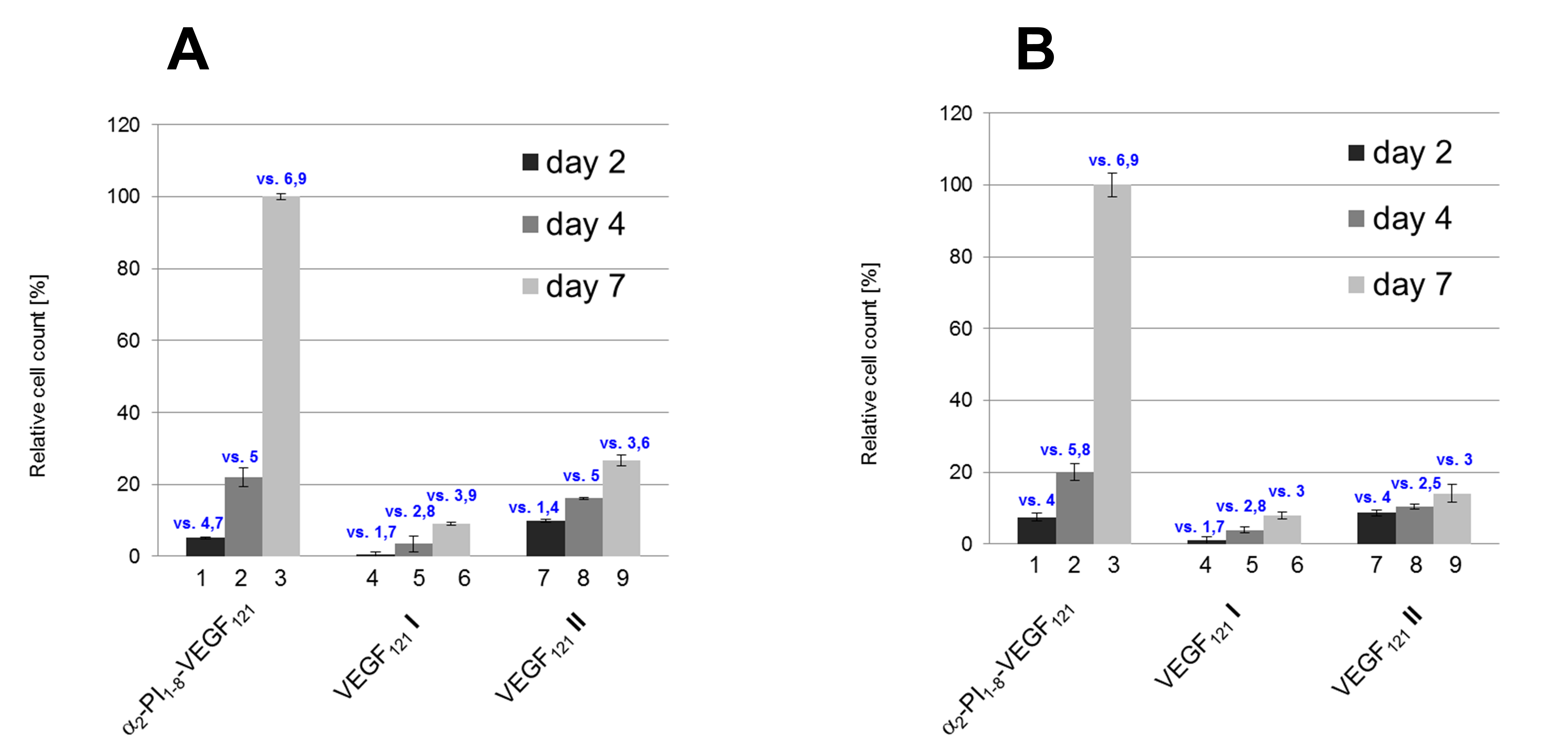

Supplement: S2 Fig — Relative cell count in a cultivation medium containing the examined VEGF121 preparations (α2-PI1-8-VEGF121 from this work, VEGF121 I expressed in E. coli, and VEGF121 II expressed in HEK cells) assessed with the fluorescent indicator resazurin after 48 hours (day 2), 96 hours (day 4) and 165 hours (day 7) of incubation, compared to the control with no VEGF (= 0%). The VEGF121 concentration in all preparations was 20 ng/mL (A) and 100 ng/mL (B). Results shown as mean ± SEM (n = 4). The statistical significance was determined by the ANOVA, Student–Newman–Keuls method; p<0.05 in comparison with the samples indicated by numbers (in blue) above the columns. (TIF) [file pone.0163697.s003.tif]
