# Supplementary material for: Enhanced Mitogenic Activity of Recombinant Human Vascular Endothelial Growth Factor VEGF121 Expressed in E. coli Origami B (DE3) with Molecular Chaperones
Source: PLoS One. 2016 Oct 7;11(10):e0163697. doi: 10.1371/journal.pone.0163697 (PMC5055331; doi:10.1371/journal.pone.0163697)
Supplement: S1 Table — Due to the risk of underestimating the potential VEGF121 concentration, we determined the VEGF121 protein concentration independently. The VEGF121 solutions were analyzed simultaneously by the FluoroProfile Protein quantification Kit and by VEGF-ELISA, in order to confirm whether VEGF-ELISA can detect VEGF in different solutions with same sensitivity. Our results suggest that VEGF-ELISA detected the VEGF in all VEGF solutions with similar sensitivity, and provided results that are 1.07–1.70 times higher than those determined by FluoroProfile Protein quantification Kit. (DOCX) [file pone.0163697.s009.docx]

| **VEGF preparation** | **Declared concentration* [µg/mL]** | **Bradford protein assay [µg/mL]** | **FPQ** [µg/mL]** | **VEGF ELISA*** [µg/mL]** | **VEGF-ELISA-output/ FPQ-output ratio** |
| --- | --- | --- | --- | --- | --- |
| α2-PI1-8-VEGF121 prepared in this work | --- | 152.0 | 108.2 | 116.0 | 1.07 |
| VEGF121 I Cat. No. CYT-343, *E. coli* expressed (Prospecbio) | 100.0 | --- | 83.9 | 100.9 | 1.20 |
| VEGF121 II Cat. No. CYT-116, HEK cells expressed (Prospecbio) | 100.0 | --- | 18.3 | 31.2 | 1.70 |

*Concentration declared by the supplier when resuspended in the recommended volume of dH_2_O or PBS

**FluoroProfile Protein Quantification Kit (Cat. No. FP0010, Sigma-Aldrich) for protein concentration determination

***VEGF121-ELISA Kit (Cat. No. KHG0112, Invitrogen)
