# Supplementary material for: Enhanced Mitogenic Activity of Recombinant Human Vascular Endothelial Growth Factor VEGF121 Expressed in E. coli Origami B (DE3) with Molecular Chaperones
Source: PLoS One. 2016 Oct 7;11(10):e0163697. doi: 10.1371/journal.pone.0163697 (PMC5055331; doi:10.1371/journal.pone.0163697)
Supplement: S1 Text — (DOC) [file pone.0163697.s012.doc]

| (OFFICEUSEONLY) |  |
| --- | --- |
| CustomerID | 捷克 |
| Host | E.coli |
| Vector | pET32A |
| CloningSites | MscI/XhoI |
| Memo |  |

Codon Optimization was done using our proprietary software with following parameters: 15% cut off was used for codon efficiency: any codon below 15% was removed except for positions with strong secondary structures (in this case codons of lower frequency were used to alleviate the problem). Secondary structure was checked using a build in M-fold module. Internal ribosomal binding sites were removed.

**Optimization Parameters**

optimizes a variety of parameters that are critical to the efficiency of gene expression, including but not

limited to:

• Codon usage bias

• GC content

• CpG dinucleotides content

• mRNA secondary structure

• Cryptic splicing sites

• Premature PolyA sites

• Internal chi sites and ribosomal binding sites

• Negative CpG islands

• RNA instability motif (ARE)

• Repeat sequences (direct repeat, reverse repeat, and Dyad repeat)

• Restriction sites that may interfere with cloning

**Optimized for your reference:**

**>1 VEGF A121 (450bp)**

tGGcCAtATGCATCATCACCACCATCACTCCTCTGGTCTGGTTCCGCGTGGCTCCGGCATGAACCAGGAACAGGTTAGCCCGCTGGCACCGATGGCGGAAGGTGGCGGTCAGAACCACCACGAAGTTGTGAAATTCATGGATGTGTACCAGCGCTCTTACTGCCACCCGATCGAAACTCTGGTGGACATCTTCCAGGAATACCCGGACGAAATCGAGTACATTTTCAAGCCGTCCTGCGTTCCGCTGATGCGTTGCGGTGGTTGCTGTAACGACGAAGGTCTGGAATGCGTGCCGACTGAAGAATCTAACATCACCATGCAGATCATGCGTATCAAACCGCACCAGGGTCAGCACATTGGTGAAATGTCTTTCCTGCAGCACAACAAATGCGAATGCCGTCCGAAAAAAGACCGTGCTCGCCAGGAAAAATGCGATAAACCGCGTCGTtgactcgag

**Aminoacid sequence:**

10 20 30 40 50 60
MSDKIIHLTD DSFDTDVLKA DGAILVDFWA EWCGPCKMIA PILDEIADEY QGKLTVAKLN

 70 80 90 100 110 120
IDQNPGTAPK YGIRGIPTLL LFKNGEVAAT KVGALSKGQL KEFLDANLAG SGSGHMHHHH

 130 140 150 160 170 180
HHSSGLVPRG SGMNQEQVSP LAPMAEGGGQ NHHEVVKFMD VYQRSYCHPI ETLVDIFQEY

 190 200 210 220 230 240
PDEIEYIFKP SCVPLMRCGG CCNDEGLECV PTEESNITMQ IMRIKPHQGQ HIGEMSFLQH

 250 260
NKCECRPKKD RARQEKCDKP RR

**
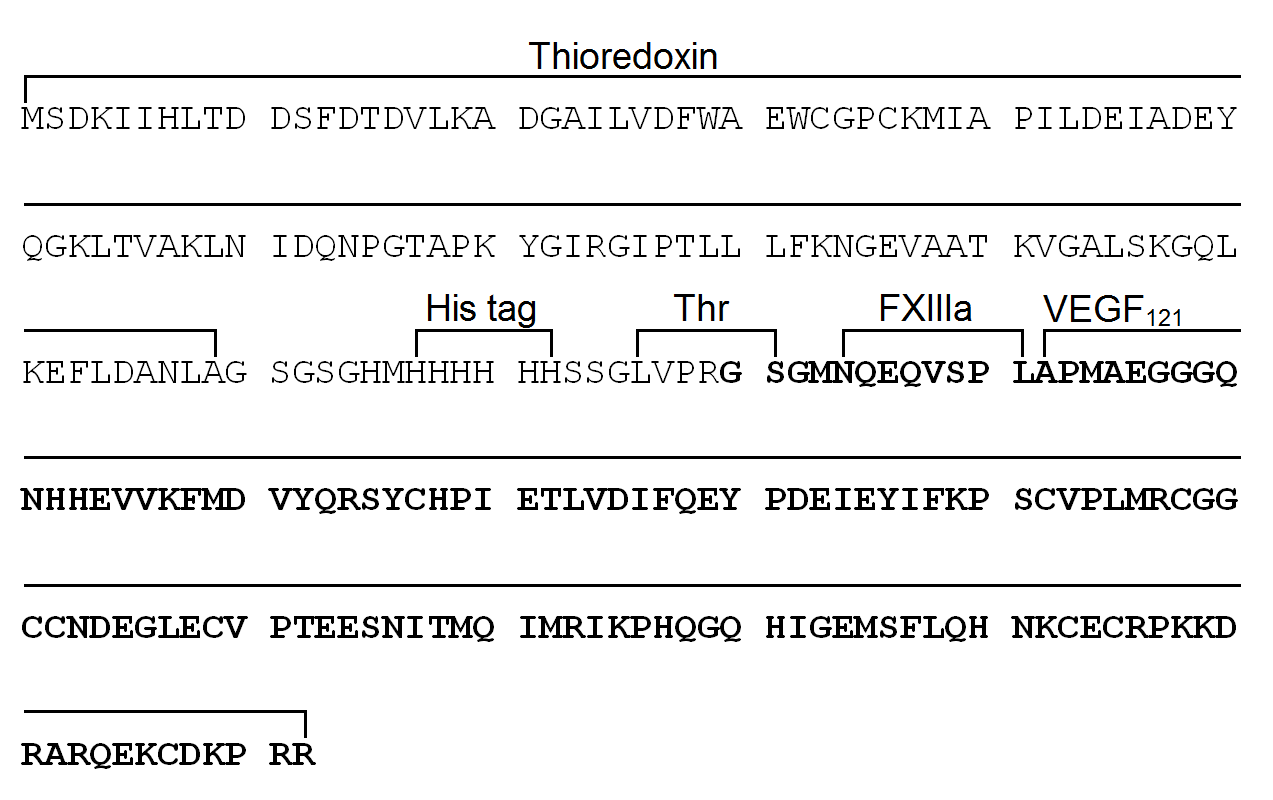
**
